# Synthetic Resveratrol Analogue, 3,3′,4,4′,5,5′-Hexahydroxy-*trans*-Stilbene, Accelerates Senescence in Peritoneal Mesothelium and Promotes Senescence-Dependent Growth of Gastrointestinal Cancers

**DOI:** 10.3390/ijms141122483

**Published:** 2013-11-14

**Authors:** Justyna Mikuła-Pietrasik, Patrycja Sosińska, Marcin Wierzchowski, Katarzyna Piwocka, Krzysztof Książek

**Affiliations:** 1 Laboratory of Gerontology, Department of Pathophysiology, Poznań University of Medical Sciences, Œwięcickiego 6 Str., Poznań 60-781, Poland; E-Mails: jmikum20@ump.edu.pl (J.M.-P.); psosinska@ump.edu.pl (P.S.); 2 Department of Chemical Technology of Drugs, Poznań University of Medical Sciences, Grunwaldzka 6, Poznań 60-780, Poland; E-Mail: mwierzch@ump.edu.pl; 3 Laboratory of Cytometry, Nencki Institute of Experimental Biology, Polish Academy of Sciences, Pasteura 3, Warsaw 02-093, Poland; E-Mail: kpiwocka@nencki.gov.pl

**Keywords:** cancer metastases, mesothelial cells, peritoneal cavity, replicative senescence, stilbenes

## Abstract

3,3′,4,4′,5,5′-Hexahydroxy-*trans*-stilbene (M8) is a synthetic resveratrol derivative, advertised as a candidate drug highly effective against numerous malignancies. Because multiple tumors prone to M8 frequently metastasize into the peritoneal cavity, this study was aimed at establishing the effect of M8 on the growth and senescence of human peritoneal mesothelial cells (HPMCs), the largest cell population within the peritoneum, actively involved in the intraperitoneal spread of cancer. The study showed that M8, used at the highest non-toxic dose of 10 μM, impairs proliferation and accelerates senescence in cultured HPMCs via an oxidative stress-dependent mechanism. At the same time, soluble factors released to the environment by HPMCs that senesced prematurely in response to M8 promoted growth of colorectal and pancreatic carcinomas *in vitro*. These findings indicate that M8 may indirectly—through the modification of normal (mesothelial) cells phenotype—facilitate an expansion of cancer cells, which challenges the postulated value of this stilbene in chemotherapy.

## 1. Introduction

Resveratrol (3,4′,5-trihydroxy-*trans*-stilbene; RVT) is a prototype stilbene widely acknowledged for its anti-ageing properties. It has been found that RVT extends lifespan in various model organisms *in vivo* [1,2] as well as delays the onset of replicative senescence (a permanent withdrawal from the cell cycle after passing a certain number of divisions [3]) in cultured cells *in vitro* [4,5]. It is believed that these effects of RVT are mainly related to an induction of NAD^+^-dependent histone deacetylases, sirtuins, which control such vital cellular processes as energy homeostasis, maintenance of genetic stability, and stress response [6]. An another aspect of the RVT story which gained tons of attention over the past decade is its tumor-suppressing activity, associated with both anti-proliferative and pro-apoptotic effects exerted towards a wide range of malignancies [7,8].

**Figure 1:**
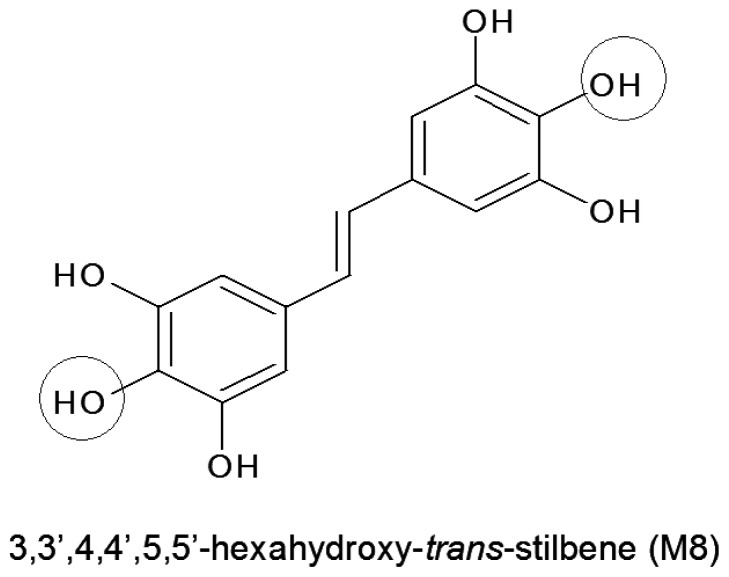
The chemical structure of stilbene M8 (3,3′,4,4′,5,5′-hexahydroxy-*trans*-stilbene). Additional hydroxy (–OH) groups in the highly reactive position *ortho* are marked in the circles. The role of the *ortho* hydroxyl groups in the augmentation of some biological properties of resveratrol (RVT) derivatives has been explained in the discussion section.

Unfortunately, due to low bioavailability and successive breakdown to less active metabolites, the clinical value of RVT is limited. In order to avoid these restrictions, numerous modified (hydroxylated or methylated) derivatives of RVT were synthesized, and their activity is now being extensively investigated [9,10]. These studies are based on a paradigm that an introduction of additional groups into the stilbene structure strengthens biological properties of RVT analogues [11]. Of these newly synthesized derivatives, high expectations are linked with 3,3′,4,4′,5,5′-hexahydroxy-*trans*-stilbene (M8) which has been found to inhibit growth and/or induce apoptosis in various malignancies, including breast and colon cancers [12,13], leukemia [14], melanoma [15], and glioma cells [16] (Figure 1).

In some cases, the anti-tumor activity of M8 appeared to be even stronger compared with RVT itself [17,18]. It is believed that this increased cytotoxicity of M8 may be attributed to the presence of the *ortho-*hydroxyl groups in its structure which localisation is supposed to amplify several biological properties of the compound. One of the properties exacerbated by the additional *ortho*-hydroxyl groups is generation of mitochondrial reactive oxygen species (ROS), especially superoxides, whose deleterious activity may underlie either anti-proliferative or pro-apoptotic capabilities of M8 [19]. It should also be noticed that in some particular situations the biological effects exerted by M8 on processes involved in cancer progression may be entirely different—in terms of their direction, not a scale—from its natural precursor. This was the case, for instance, for the angiogenic behaviour of endothelial cells which upon exposure to M8 displayed increased effectiveness of proliferation and migration, conversely to RVT which inhibited both the processes [20].

The majority of cancers whose expansion was inhibited by M8 frequently metastasize into the peritoneal cavity [21,22], in which their further spread is controlled by a crosstalk with normal peritoneal mesothelium (HPMCs) [23]. As shown recently, the intraperitoneal cancer progression is facilitated by senescent HPMCs [24,25], which accumulate in the peritoneum *in vivo* [26].

Because M8 is advertised as a promising candidate for therapy of certain (also peritoneum-associated) tumors [13,27] whose expansion usually increases with age, the goal of our study was to examine the effect of this stilbene on biological properties of HPMCs, particularly their proliferative potential and replicative senescence. In addition, we established a co-culture system in which we assessed whether soluble factors released to the environment by HPMCs subjected to M8 may modulate growth capabilities of ovarian, colorectal and pancreatic carcinomas.

## 2. Results and Discussions

### 2.1. Stilbene M8 (3,3′,4,4′,5,5′-Hexahydroxy-*trans*-Stilbene) Impairs Viability of HPMCs (Human Peritoneal Mesothelial Cells) at the Doses Higher than 10 μM

**Figure 2:**
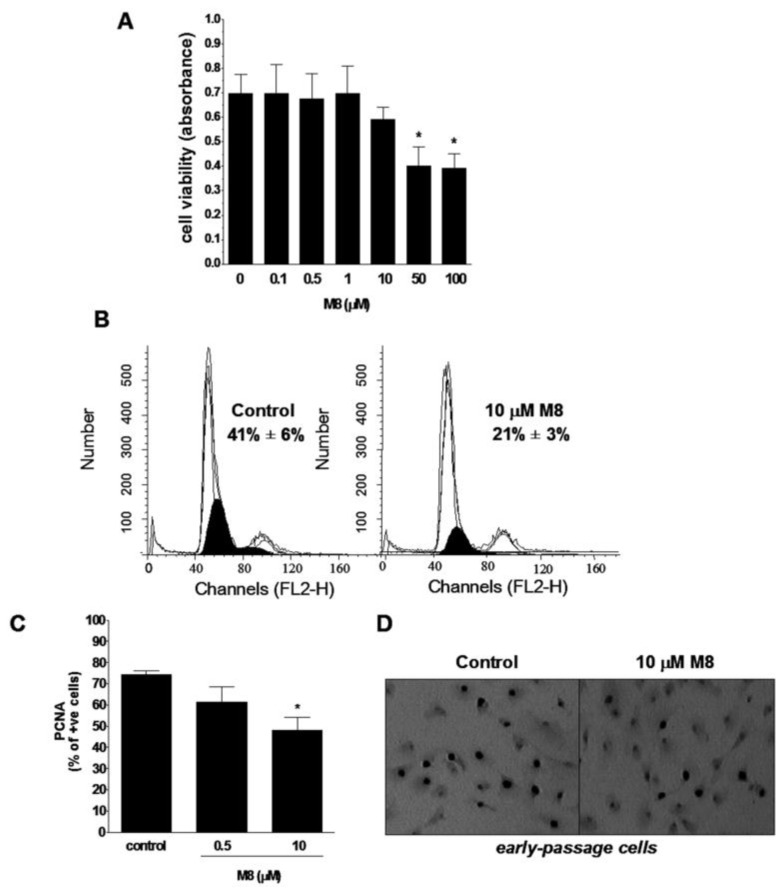
Effect of M8 on viability and proliferation of early-passage human peritoneal mesothelial cells (HPMCs). Cell viability was examined using the MTT assay on the confluent cultures (**A**); Cell proliferative capacity was examined according to the measurements of cell distribution within the cell cycle, in particular in the S phase (darkened area) (**B**); and the percentage of cells positive for proliferating cell nuclear antigen, PCNA (**C**); The values shown in the panel B indicate the size of cell fraction in the S phase, in the control cells and the cultures exposed to 10 μM M8, respectively. Representative results of PCNA immunostaining in the control cells and the cultures exposed to 10 μM M8 (**D**). PCNA-positive cells exhibit brown nuclei. All experiments were performed on cell cultures derived from the first passage and exposed to M8 at 0.5 and 10 μM for 24 h. The asterisks indicate a significant difference compared to the control group. Experiments were performed in triplicates with HPMC (human peritoneal mesothelial cells) cultures derived from 8–12 different donors.

In order to examine the effect of resveratrol analogue M8 on HPMC viability, confluent early-passage cells were exposed for 24 h to M8 in wide range of doses (from 0.1 to 100 μM), and then the MTT [3-(4,5-dimethylthiazol-2-yl)-2,5-diphenyltetrazolium bromide] reduction assay was performed. The experiments showed that stilbene M8 is non-toxic for HPMCs up to the concentration of 10 μM. At the greatest doses of M8, 50 and 100 μM, a significant cytotoxicity was recorded (Figure 2A). Because the concentration of 10 μM appeared to be a threshold for sustained viability of HPMCs upon exposure to M8, additional tests were performed in which the cells were treated with this stilbene for seven days. Also in this case, however, the viability of cells exposed to M8 did not alter significantly from the values obtained for the control group (data not shown).

Accordingly, two final concentrations of M8 were chosen for all subsequent experiments. The first dose was 0.5 μM which from one hand was entirely safe for HPMCs, and from the other hand appeared to efficiently stimulate growth and delay replicative senescence in HPMCs exposed to M8 precursor, resveratrol [5]. The second concentration was 10 μM which seemed to be the safest for HPMCs in short-term and chronic experiments.

### 2.2. Stilbene M8 Inhibits Proliferation of Early-Passage HPMCs

The impact of the stilbene M8 on cell proliferative capacity was assessed in short-term experiments using low-density cultures of early-passage HPMCs. As a measure of growth capabilities, the fraction of DNA replicating cells in the S phase of the mitotic cycle and the percentage of cells expressing the S-phase specific proliferating cell nuclear antigen (PCNA) were quantified. The studies showed that 0.5 μM M8 does not affect both the parameters associated with reproducibility of young HPMCs. At the same time, 10 μM M8 reduced the magnitude of either the pool of cells in the S phase of the cell cycle (Figure 2B) or the subset of cells positive for PCNA antigen (Figure 2C,D).

### 2.3. Stilbene M8 Accelerates Senescence of HPMCs

**Figure 3:**
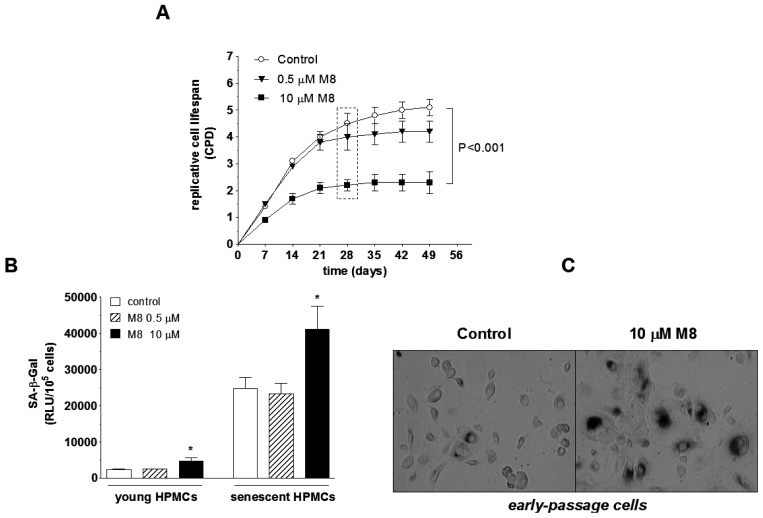
Effect of M8 on replicative senescence of HPMCs. Cells were forced to senescence by serial passaging at seven-day intervals, as described in the methods section, and then the cumulative number of population doublings (CPD) (**A**) and the activity of SA-β-Gal (**B**) were examined. A box in the panel (**A**) indicates the time-point at which M8-treated cells became senescent while the control cells still proliferated vigorously. The samples of conditioned medium were taken at this point and then used in the experiments shown in Figure 4. Representative results of early-passage HPMC staining for SA-β-Gal (positive cells have *dark colour* within the cytoplasm) (**C**). The asterisks indicate a significant difference compared to the control group. Experiments were performed in with HPMC cultures derived from 12 different donors.

In order to answer the question of whether M8 affects the replicative lifespan of HPMCs, the cells were sub-cultivated at fixed seven-day intervals until complete cessation of their capacity to divide. Under such a regimen, the expandability of cells exposed to 0.5 μM M8 was comparable to the control group. At the same time, when the cells were continuously propagated in the presence of 10 μM M8, their lifespan was reduced by a half (Figure 3A).

Rapid loss of cell ability to replicate and resulting premature entry into senescence of cells exposed 10 μM M8 corresponded to increased activity of an indicatory enzyme for senescent cells, SA-β-Gal. Interestingly, up-regulated SA-β-Gal in cells subjected to 10 μM M8 was also recorded in early-passage cultures (Figure 3B,C).

### 2.4. Stilbene M8 Induces Oxidative Stress in Early-Passage and Senescent HPMCs

**Figure 4:**
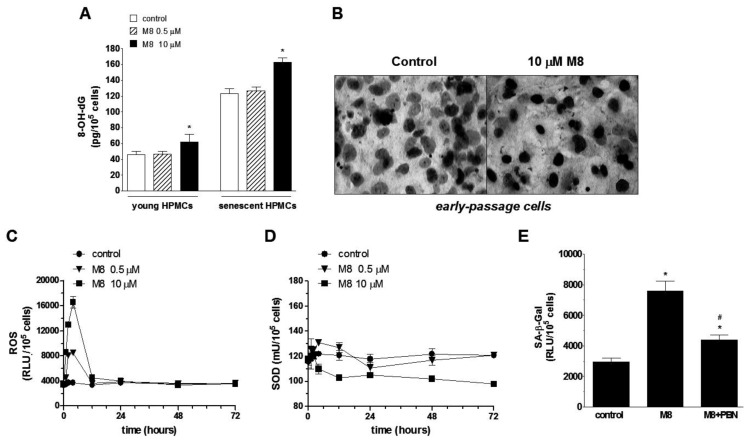
The role of oxidative stress in M8-dependent premature senescence of HPMCs. The changes in 8-OHdG concentration in early-passage and senescent HPMCs subjected to M8 (**A**) as well as representative results of 8-OHdG immunostaining (positive cells have *dark nuclei*) in early-passage cultures (**B**); The results of time-course experiments for reactive oxygen species (ROS) production (**C**) and superoxide dismutase (SOD) activity (**D**) in early-passage cells exposed to M8; The effect of ROS scavenger, *N*-*tert*-butyl-α-phenylnitrone (PBN, at 800 μM), on the activity of SA-β-Gal in cells treated with 10 μM M8 (**E**). The cells were plated at a low density and then subjected to the stilbene for up to 72 h. The asterisks indicate a significant difference compared to the control group while the symbol # indicates a significant difference compared with M8-treated cells. Experiments were performed in duplicates with HPMCs derived from 8–10 different donors.

Because senescence is considered as cell response to excessive DNA injury, a quantitative analysis of concentration of 8-OHdG (a major product of DNA oxidation) in cells subjected to M8 was performed. The results showed that the magnitude of oxidative DNA damage in cells treated with 0.5 μM M8 remains unchanged. Conversely, in cells subjected to 10 μM M8, concentration of 8-OHdG was significantly higher compared with untreated control cells in both young and senescent cultures (Figure 4A,B).

Afterwards, the time-course experiments on early-passage cells (derived from the first passage) were performed in order to examine the effect of M8 on two aspects of cellular oxidative stress; that is generation and neutralization [SOD (superoxide dismutase)-dependent] of reactive oxygen species (ROS). The experiments showed that cells subjected to M8 released ROS time-dependently with the highest value reached after the first 4 h of incubation. The magnitude of ROS production in cells exposed to 10 μM M8 was at that moment twice as high as in cells exposed to 0.5 μM stilbene. After reaching the peak value, ROS level rapidly declined and reached the baseline level at the 12 h of the experiment for the both concentrations of M8 (Figure 4C).

The simultaneous assessment of SOD activity revealed that in the cells subjected to 0.5 μM M8, after an elevation period taking 12 h (the peak at the 4 h), it steadily decreased reaching the initial level. Conversely, in cells exposed to 10 μM M8, SOD declined from the very beginning so that at the end of exposure it was substantially lower compared to the baseline values (Figure 4D).

In order to confirm if oxidative stress mediates premature senescence of HPMCs treated with M8, early-passage cells were exposed for 72 h to standard medium, to medium with 10 μM M8, and to medium with 10 μM M8 enriched in a spin-trap ROS scavenger, *N*-*tert*-butyl-α-phenylnitrone (PBN), used in the concentration of 800 μM [26]. Afterwards, the activity of senescence marker SA-β-Gal was examined, and the results showed that the neutralization of ROS by simultaneous incubation with PBN significantly attenuated M8-mediated increase in SA-β-Gal activity (Figure 4E).

### 2.5. Stilbene M8 Promotes Senescent HPMC-Dependent Growth of Cancer Cells

Samples of conditioned medium (CM) harvested from HPMCs treated with M8 (at senescence-promoting dose of 10 μM) were used to examine growth response of cancer cells, metastasizing into peritoneal cavity. These included ovarian cancer (A2780), colorectal cancer (SW480), and pancreatic cancer (PSN-1). In these studies, CM from HPMCs propagated in standard growth medium collected at the time-point corresponding to senescence of M8-treated cells was used as the control. Cancer cell proliferation was assessed using the [^3^H]-thymidine uptake assay, indicating the efficiency of DNA replication.

**Figure 5:**
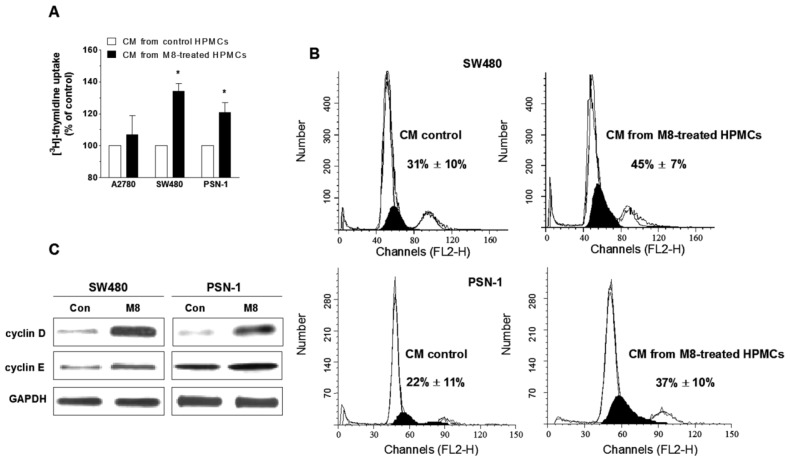
Effect of M8 on HPMC-dependent proliferation of ovarian (A2780), colorectal (SW480), and pancreatic (PSN-1) cancer cells. Cell proliferation was assessed on low-density cultures exposed for 24 h to the conditioned medium harvested from HPMCs that senesced prematurely in response to 10 μM M8 and to the medium from the control cells (from the same passage), using [^3^H]-thymidine incorporation assay (**A**); The measurements of cancer cell distribution within the cell cycle, in particular in the S phase (darkened area) (**B**); The values shown in the panel (**B**) indicate the size of cell fraction in the S phase, in the cultures exposed to CM (conditioned medium) from control HPMCs and to CM from HPMCs prematurely senesced in response to 10 μM M8, respectively. The representative results of western-blot analysis of cell cycle regulators: cyclins D and E. Bands corresponding to target molecules were compared to those for GAPDH (**C**). The asterisks indicate a significant difference compared to the control cells treated as 100%. The experiments were performed in quadruplicates with CM from HPMC cultures obtained from 5 different donors.

The experiments showed that both cancers of gastrointestinal origin exposed to CM from HPMCs that senesced prematurely under exposure to M8 displayed markedly improved DNA synthesis. In contrast, the proliferative behavior of ovarian cancer cells remained unchanged (Figure 5A). An increased efficacy of DNA replication in response to CM from M8-treated HPMCs also confirmed an analysis of cell distribution in the cell cycle, in particular the size of the fraction of cells in the S phase (Figure 5B). Further analysis of cell cycle positive regulators, cyclin D and cyclin E, using western-blot technique, revealed that cancer cell growth acceleration upon 10 μM M8 was associated with significantly up-regulated expression of both G_1_ phase-specific cyclins (Figure 5C).

### 2.6. Discussion

A supreme anti-cancer drug should effectively stop an expansion of malignant cells without exerting any negative impact on biological properties of nearby normal cells. Most frequently, however, these bystanders become victims of chemotherapy which is usually linked with high doses of drugs which have to be used in order to guarantee a desirable effect. On the other hand, current studies aimed at establishing new classes of anti-cancer agents often skip (or at least underestimate) the crucial issue of a drug safety toward normal cells in regions encompassed by pathology. In some cases, such a simplistic approach may exert a paradoxical effect leading to enhanced tumor progression [28].

In this paper, we examined the effect of the synthetic resveratrol analogue M8 (recently postulated as an effective agent in a fight against multiple kinds of cancer cells [13,16,27]) on viability and proliferation of normal human peritoneal mesothelial cells (HPMCs) *in vitro*. The choice of HPMCs as an experimental model was dictated by the fact that the majority of carcinomas which appeared to be vulnerable to M8 has been found to disseminate in the peritoneal cavity, often in a HPMC-dependent mechanism [29–31]. Moreover, it has recently been shown that synthetic resveratrol derivatives, including M8, stimulate—in contrast to their natural precursor—HPMC-dependent angiogenesis [20], and thus may contribute to the development of a permissive microenvironment for intraperitoneal cancer spread.

Our current research showed that M8 is non-toxic for HPMCs only when it is used at a concentration up to 10 μM. At the same time, the measurements of young cell proliferation revealed that HPMC exposure to 10 μM M8 markedly inhibited their growth (a lower concentration had no effect), as we evidenced analyzing the size of DNA-replicating cell fraction in the S phase of cell cycle and the subset of cells expressing S phase-specific proliferative marker, PCNA [32]. It must be stressed, however, that the dose of 10 μM is relatively low, especially in the context of multiple studies on malignant cells in which M8 inhibited cell growth and/or triggered apoptosis. For instance, the proliferation assays performed on various types of breast cancer cells treated with M8 revealed that IC_50_ values for this stilbene range from 90 to 127 μM [12]. In studies on melanoma cells, IC_50_ was between 20 and 25 μM [15]. Similar tendency was reported in the case of apoptotic potency of M8 which achieved the values near to maximal (100%) at the doses from 12.5 to 100 μM [13].

Another aspect of HPMC behavior examined under exposure to M8 was cellular senescence. This process is of special importance in the context of cancer adhesion-promoting activity of senescent HPMCs which was recently documented with respect to ovarian, colorectal, and pancreatic cancers [24,25]. Here, we were able to show that M8 used at 10 μM markedly shortens a long-term HPMC expandability which results in their premature entry into senescence (decreased number of divisions combined with increased activity of senescence marker, SA-β-Gal).

Because replicative senescence of HPMCs is primarily regulated by deleterious activity of ROS [26,33], we examined of whether M8 may accelerate an exhaustion of cell capacity to divide via increased oxidative stress. The experiments showed that, indeed, M8 markedly increases oxidative stress in HPMCs, as evidenced according to increased levels of oxidative DNA damage (8-OHdG) in early-passage and senescent HPMCs. This may be, in turn, attributed to an imbalance between up-regulated generation of ROS and decreased activity of antioxidative enzyme, SOD. An interesting issue is that ROS that peaked in the cells treated with M8 barely after few hours of exposure (and then returned to its baseline level) exerted its pro-senescence activity several days later which is in line with our previous observations that premature HPMC growth cessation depends to the largest extent on the deleterious activity of ROS (mainly DNA injury) experienced by the cells at the very early stages of their replicative history [26].

When it comes to an excessive ROS release in HPMCs subjected to M8, it is probably associated with the presence of two additional hydroxyl groups in a highly reactive position *ortho* in the stilbene structure (see Figure 1). The studies employing a microsomal model showed that the *ortho* compounds are able to form the cytotoxic semiquinones and thus stimulate additional oxygen consumption. This is possibly performed via redox cycling at expense of reducing equivalents transferred by cytochrome b5 and leads to augmented generation of ROS [19]. This scenario confirmed our previous studies which showed that the magnitude of ROS production by young and senescent HPMC upon treatment with M8 was significantly higher compared with other stilbenes possessing lower number of the *ortho* hydroxyl groups or not possessing them at all [20]. As for an insufficient antioxidative protection, our results are in keeping with those obtained by other groups and showing decreased activity of SOD and catalase in breast cancer cells treated with M8 [12]. The causative involvement of oxidative stress in premature senescence of HPMCs upon exposure to M8 confirmed experiments in which activity of senescence marker SA-β-Gal in cells exposed to M8 was significantly attenuated in response to cell protection against ROS using the spin-trap scavenger PBN.

In order to verify a scenario in which HPMCs that senesced prematurely in response to M8 may facilitate cancer cell expansion, we used three cancer cell types whose adhesion was promoted by senescent HPMCs [24,25], and examined their growth response to soluble factors released to the environment by M8-treated HPMCs using M8- and serum-deprived conditioned medium. The results showed that the growth capabilities of colorectal and pancreatic (but not ovarian) carcinomas were markedly enhanced compared with the cells exposed to the medium from control cultures from the same passage. This, in turn, proves that the pro-senescence effect of M8 observed *in vitro* may be biologically relevant and may cause an improvement of HPMC-dependent progression of malignancy. From the mechanistic point of view, cancer growth acceleration was associated with an up-regulation of proteins positively controlling progression of cell cycle from the G_1_ to S phase, namely cyclins D and E which was eventually reflected by increased fraction of cells in the S phase of cell cycle.

As for soluble HPMC-derived mediators of this phenomenon, VEGF and IL-8 may play a causative role, as they have an ability to directly or indirectly facilitate cancer cell proliferation [34,35], and their production by senescent HPMCs treated with M8 was markedly increased [20]. At the same time, it is puzzling why the conditioned medium obtained from HPMCs treated with M8 promoted exclusively the growth of gastrointestinal cancers but failed to affect proliferation of ovarian carcinoma. One may only speculate that this lack of effect may result from the different mechanisms of the reciprocal interactions between senescent HPMCs and those types of cancers, which have previously been documented with respect to their adhesion [24,25]. In addition, it cannot be ruled out that the effect we observed may be cell type-specific and related, e.g., to the differences in the expression of surface molecules between certain types of ovarian cancer cells [24,36]. Regardless, the exact reason for these differences should be identified.

## 3. Experimental Section

### 3.1. Chemicals

Unless otherwise stated, all chemicals and plastics were purchased from Sigma-Aldrich Corp. (St. Louis, MO, USA). 3,3′,4,4′,5,5′-hexahydroxy-*trans*-stilbene (M8) was synthesized in the Institute of Pharmaceutical Technology at the Poznań University of Medical Sciences (Poznań, Poland), using standard chemical procedures [37]. Stock solution of M8 was prepared in dimethyl sulfoxide (DMSO) and diluted in culture medium to desired final dose.

### 3.2. Cell Cultures

Human peritoneal mesothelial cells (HPMCs) were isolated from the pieces of omentum. The tissues were obtained from consenting patients undergoing abdominal surgery. The study was approved by the institutional ethics committee. The cells were propagated in M199 medium enriched with l-glutamine (2 mM), penicillin (100 U/mL), streptomycin (100 μg/mL), and 10% fetal bovine serum (FBS). During the experiments, the cells were continuously exposed to standard medium (control) and to the medium supplemented with M8 (at indicated doses). The culture media were exchanged every three days. Senescence of HPMCs was induced by serial passaging until exhaustion of cell replicative capacity. Cultures were considered “senescent” when they stopped divide, displayed hypertrophic morphology, and more than 70% of the population stained positively for SA-β-Gal [26]. Cells harvested after seven days of incubation (during first passage) were considered “young”.

Ovarian cancer cells (A2780) and pancreatic cancer cells (PSN-1) were obtained from the European Collection of Cell Cultures (Porton Down, UK). Colorectal cancer cell line SW480 was purchased from the American Type Culture Collection (Rockville, MD, USA). All cancers were propagated in RPMI-1640 medium supplemented with 10% fetal bovine serum (FBS).

### 3.3. Determination of Cell Viability

Cells were seeded into 96-well plates at a high density of 1 × 10^5^ cells/cm^2^ and allowed to attach for 16 h. Then cells were incubated in medium containing 1.25 mg/mL of the MTT salt [3-(4,5-dimethylthiazol-2-yl)-2,5-diphenyltetrazolium bromide] for 4 h at 37 °C. The formazan product generated was solubilized with 20% sodium dodecyl sulphate and 50% *N*,*N*-dimethylformamide. Absorbance of the converted dye was recorded at 595 nm with a reference wavelength of 690 nm.

### 3.4. Cell Cycle Analysis

Cells were harvested with trypsin-EDTA solution and fixed in ice-cold 70% ethanol overnight at −20 °C. After washing with PBS, cells were re-suspended in 0.1 M sodium citrate, pH 7.8 for 1 min, and incubated for 30 min in PBS containing 5 mg/mL of propidium iodide (Molecular Probes, Eugene, OR, USA) and 0.1 mg/mL of RNase A. In order to determine cell distribution in the cell-cycle, one million cells was analyzed using a FACSCalibur™ flow cytometer with ModFit LT™ software (Verity Software House, Topsham, ME, USA).

### 3.5. Immunocytochemistry for Proliferating Cell Nuclear Antigen (PCNA)

HPMCs were cultured in Lab-Tek™ Chamber Slides (Nunc, Roskilde, Denmark), fixed with 70% ethanol, and probed with specific anti-PCNA monoclonal antibody (clone PC10; Dako, Glostrup, Denmark), diluted 1:500. Bound antibodies were detected by immunoperoxidase staining using the EnVision System (Dako).

### 3.6. Detection of Senescence-Associated β-Galactosidase (SA-β-Gal)

Activity of SA-β-Gal was quantified in cell extracts by measuring the rate of conversion of 4-methylumbelliferyl-β-d-galactopiranose to 4-methylumbelliferone, and recorded using spectrofluorometer Wallac Victor 2 (Perkin-Elmer, Waltham, MA, USA) with excitation at 360 nm and emission at 465 nm. Expression of SA-β-Gal was also visualized cytochemically upon exposure (for 6 h at 37 °C) to 1 mg/mL 5-bromo-4-chloro-3-indolyl-β-d-galactopyranoside (X-Gal), 5 mM potassium ferrocyanide, 5 mM potassium ferricyanide, 150 mM NaCl, 2 mM MgCl_2_ and 40 mM citric acid, at pH 6.0.

### 3.7. Measurements of Oxidative Stress-Related Parameters

Production of reactive oxygen species (ROS) was assessed in cells probed with a fluorescent dye 2′,7′-dichlorodihydrofluorescein diacetate (H_2_DCFDA, Molecular Probes). The fluorescence generated was monitored in a spectrofluorometer Wallac Victor 2 (Perkin-Elmer) with excitation at 485 nm and emission at 535 nm. Activity of SOD was measured in cell extracts using a commercially available kit (R&D Systems Europe, Abingdon, UK). Concentration of 8-hydroxy-2′-deoxyguanosine (8-OHdG) was assessed with a competitive immunoassay (Cell Biolabs Inc., San Diego, CA, USA). In some experiments, 8-OHdG was visualized immunocytochemically with monoclonal anti-8-OHdG antibody (Trevigen, Gaithersburg, MD, USA), diluted 1:300.

### 3.8. HPMC-Derived Conditioned Medium

Samples of conditioned media (CM) were harvested from HPMC cultures reaching senescence in the presence of M8, and from the corresponding control cells at the same passage. Briefly, when the cells exposed to M8 became senescent, both groups of cells were carefully washed with PBS to remove M8 and the serum, trypsinized, and counted. Afterwards 3 × 10^5^ cells was seeded into 25 cm^3^ flasks, allowed to attach for 4 h, and incubated in fresh serum-free medium for 72 h. The samples of CM collected were centrifuged, filtered through a 0.2 μm pore size filter to remove any cellular debris, and then stored in aliquots at −80 °C until required.

### 3.9. Cancer Cell Proliferation Measurements

Cancer cell proliferation was examined according to an incorporation of [^3^H]-thymidine into DNA of dividing cells. Briefly, cancer cells were plated onto 48-well clusters at a density of 1.5 × 10^3^ cells per well and allowed to attach for 2 h. Then, the cells were growth synchronized by serum deprivation for 4 h. Next, cells were exposed to HPMC-derived conditioned media for 24 h in the presence of [^3^H]-thymidine (as methyl-[^3^H]-thymidine; 1 μCi/mL; Institute of Radioisotopes, Prague, Czech Republic). After the incubation the cells were harvested with a trypsin-EDTA (0.05%–0.02%) solution and precipitated with 10% (*w/v*) trichloroacetic acid (TCA). The precipitate was dissolved in 0.1 N NaOH and the released radioactivity was measured in a β liquid scintillation counter (Wallac, Perkin Elmer, Warsaw, Poland). The results obtained were normalized per number of HPMCs that gave rise to the conditioned medium at a particular passage [38]. In order to sensitize cancer cells to HPMC-specific conditioned medium, A2780, SW480 and PSN-1 cells were exposed to M199 medium for two weeks before the scheduled experiment.

### 3.10. Western Blotting for Cell Cycle Regulators

Cells were scraped on ice, lysed in a buffer containing 20 mM Tris-HCl at pH 7.3, 200 mM NaCl, 1 mM EDTA, 1% Triton X-100, 1 mM PMSF, and protease inhibitor cocktail (Roche Diagnostics, Mannheim, Germany), and homogenized by sonication. Samples corresponding to 40 μg protein were resolved by 15% SDS-PAGE and transferred to Immobilon-P polyvinylidene difluoride membranes (Millipore, Eschborn, Germany). The membranes were blocked with 5% non-fat powdered milk and probed with antibodies against either p16, p21, p53, cyclin D, cyclin E or GAPDH (Santa Cruz Biotechnology, Santa Cruz, CA, USA). Bound antibodies were visualized following incubation with the peroxidase-labeled secondary antibodies (Dako) and the exposure to chemiluminescence reagent (ECL; Amersham Pharmacia Biotech, Castle Hill, Australia).

### 3.11. Statistics

Statistical analysis was performed using GraphPad Prism™ 5.00 software (GraphPad Software, San Diego, CA, USA). The means were compared with repeated measures analysis of variance (ANOVA) with the Newman-Keuls test as *post-hoc*. When appropriate, the two-way ANOVA and Wilcoxon matched pairs test were used. Results were expressed as means ± SEM. Differences with a *p*-value < 0.05 were considered to be statistically significant.

## 4. Conclusions

Altogether, our results show that the clinical usefulness of the stilbene M8 in the anti-cancer therapy is seriously jeopardized by its HPMC senescence-promoting activity. In addition, they justify a verification of any promising effects obtained for anti-cancer drug candidates using normal nearby cells which, under certain conditions, may exert paradoxical cancer-promoting activity.
